# Z2F: Heterogeneous graph-based Android malware detection

**DOI:** 10.1371/journal.pone.0300975

**Published:** 2024-03-28

**Authors:** Ziwei Ma, Nurbor Luktarhan

**Affiliations:** Xinjiang Multilingual Information Technology Research Center, Xinjiang University, Urumqi, China; Prince Mohammad Bin Fahd University, SAUDI ARABIA

## Abstract

Android malware is becoming more common, and its invasion of smart devices has brought immeasurable losses to people’s lives. Most existing Android malware detection methods extract Android features from the original application files without considering the high-order hidden information behind them, but these hidden information can reflect malicious behaviors. To solve this problem, this paper proposes Z2F, a detection framework based on multidimensional Android feature extraction and graph neural networks for Android applications. Z2F first extracts seven types of Android features from the original Android application and then embeds them into a heterogeneous graph. On this basis, we design 12 kinds of meta-structures to analyze different semantic spaces of heterogeneous graphs, mine high-order hidden semantic information, and adopt a multi-layer graph attention mechanism to iteratively embed and update information. In this paper, a total of 14429 Android applications were detected and 1039726 Android features were extracted, with a detection accuracy of 99.7%.

## 1 Introduction

With the development of technology, mobile smart devices are increasingly connected to people’s lives. In order to satisfy the various needs of people’s lives and production, a wide range of mobile applications have been developed. According to research, Google’s Android system dominate smartphone operating systems. The open-source nature of the Android system is like a double-edged sword. While maximizing convenience for developers, it also faces a great danger of intrusion by malicious elements. The number of mobile applications is increasing rapidly, which has led to the entry of malicious applications that invade smart devices and steal users’ private information, thus causing harm to users such as the leakage of important data and financial losses. Mobile applications are so relevant to people’s lives, and the detection of malware is urgent.

Conventionally, researchers use static analysis [1] or dynamic analysis [2] methods for Android malware detection. The static analysis first uses decompilation tools to decompile the Android application installation package files, generating a series of decompiled files consistent with the original development framework, reading the decompiled files, then extracting predefined Android features for detection. The dynamic analysis needs to use a sandbox to isolate the environment, running programs on real devices or emulators while extracting behavioral data and then determine whether the application is benign or malicious. Dynamic analysis is costly in time and manpower, static analysis cannot resist code obfuscation. To solve these problems, detection methods based on machine learning [3–11] are gradually emerging, which use feature engineering to obtain key malware features and represent it in the form of vectors, followed by classification algorithms to classify Android applications as benign or malignant. However, these methods often ignore the high-level hidden information behind the key characteristics of Android, which malware developers want to hide. Therefore, it is necessary to find an efficient and fast method to extract these hidden information from the key characteristics of Android, so that the Android malware detection task can perform better in terms of performance.

Graph neural network technology used to model rich relationships between different elements is used in many fields [12–16], heterogeneous graph networks(HIN) [17] as a emerging graph neural network quickly caught people’s attention. It brakes through the single type of nodes and edges in the homogeneous graph and can accommodate data of different types, which provide a richer and more comprehensive description of entity relationships in the real world. Learning based on heterogeneous graphs enables the discovery of implicit associations, which can reveal hidden association information between different nodes. In the field of Android application detection, we extract Android features, embedding in heterogeneous graphs and then discover hidden correlations among them. However, learning based on a heterogeneous graph is not an easy task, and the diversity of its node and edge types poses great difficulties. Although Yiming Hei [18] has solved this problem by defining different meta-structure templates and using edge attention mechanisms to learn heterogeneous graph data, it does not consider the impact of redundant features, resulting in the problem of causing detection performance to be less than optimal.

In this paper, we propose Z2F, a heterogeneous graph-based malware detection framework for Android. Z2F is aimed at exploring the hidden information behind the multidimensional Android features. By embedding them into the heterogeneous graph and mining the heterogeneous graph data from different semantic spaces to obtain the hidden relationships, using these hidden information to better distinguish malware from benign. Under extensive research and experts advice, we first use Apktool [19] to decompile the Android application installation package file and extract the permission, API, package, class, processor, and interface from the decompiled Android Manifest.xml file, .smali files, .so files, combined with the official documents provided by Google to get the permission group information. Building heterogeneous graphs of applications, combining domain expert knowledge and extensive research, 12 different meta-structures [20] are designed, including 5 metagraphs and 7 metapaths, for exploring hidden information of heterogeneous graphs in different semantic spaces.

Z2F uses one-hot encoding to represent original text features and embeds them into the heterogeneous graph. After the feature vectors are iteratively updated and aggregated through the multi-layer attention mechanism [21] based on meta-structures, the final numerical embeddings are fed into a classifier for detection. First, Android features are embedded, and then for each meta-structure, we use the graph attention mechanism [22] to obtain the contribution of neighbor nodes to the central node so as to perform the first node update. Then use the support vector machine to learn and get every meta-structure’s contribution rate, choosing significant meta-structures and aggregating them with graph attention mechanism using learned weights to get the final node embedding. This enables the updated node information to preserve diverse semantic details to the fullest extent, making model training more efficient and lightweight. The experiments show that deep semantic information mining of heterogeneous graphs for Android features is significantly helpful in Android malware detection, Z2F outperforms all baselines in terms of accuracy, indicating its effectiveness and reliable for Android malware detection. We make Z2F publicly available at:https://github.com/Jully-xiaoman/Z2F.

This paper makes the following contributions:

1. With the help of AndroZoo [23], We collected and decompiled 20,000 Android applications, checking 14,429 Android applications. The large and diverse sample base ensures the richness, extensiveness, and authenticity of the experimental data from the root, providing strong support for extraction and exploration.
2. This article proposes after use one-hot encoding of text-type Android features, and then use the Linear Discriminant Analysis(LDA) algorithm to reduce the dimensionality of the Android features to remove redundant information, thereby improving detection performance.
3. This paper designs 12 meta-structures to obtain hidden information behind features. In order to maximize accuracy and simplify the description of Android application behaviors, we use a selection mechanism to aggregate hidden information. The result shows that the hidden information provide strong support for the detection of Android applications.

Organization: Part 2 shows the literature survey for the study. Part 3 shows our proposed malware detection methodology in detail and core techniques. The fourth section describes the creation process of our dataset and uses visualization methods to provide readers with a macro overview of the dataset. Part 5 presents the experimental results and conducted some discussions on the results. In part 6, the work of this article is summarized and future work is prospected.

## 2 Literature survey

The general idea of the Android malware detection method based on traditional feature engineering is to first manually extract Android features, perform feature engineering on the extracted Android features, and then classification. The extracted Android features are divided into static Android features and dynamic Android features. Static Android features mainly include permissions, API functions, and intent. These features completely record the behavior of the application, such as calling device functions and accessing user information. William Enck [24] et al. used a variant of security requirements engineering technology to conduct in-depth security analysis on Android and generated 9 permission-based malware detection rules, which were matched when installing applications to distinguish benign and malignant applications. Seung-Hyun Seo [25] in their proposed analysis tool DroidAnalyzer, the most commonly used risk APIs were used to identify Android malware, and official applications were reviewed and screened using this tool. Ali Feizollah [26] evaluated the effectiveness of Android intents as salient features for identifying malicious applications, and compared them with methods that use permissions to identify malicious applications. The experimental results showed that the richness of intents semantic relationships enable better encoding of malware.

Dynamic Android features mainly refer to the behavior of applications during running. Vikas Sihag [27] used the behavioral characteristics of dynamic analysis of applications executed in a simulated environment to detect 13,553 different types of applications, with a detection rate of 98.08%. Pengbin Feng [28] proposed an effective dynamic analysis framework, called EnDroid, which obtains multiple types of dynamic behavior characteristics through system-level behavior tracking, thereby achieving high-precision malware detection. Arvind Mahindru [29] analyzed 11,000 Android application packages from various fields, extracted a set of 123 dynamic permissions, and used 5 machine learning classification algorithms for classification, and its detection achieved satisfactory results.

Static detection is faster, but is easily affected by code obfuscation, resulting in a high false positive rate. Dynamic detection requires real-time monitoring, so the overhead is large and it cannot capture all malicious behaviors.

In graph-based Android application detection, Marwan Omar [30] used his own graph convolutional neural network as a baseline, combined with expert data science methods, and finally achieved 99.183% accuracy in Android malware detection by continuously fine-tuning the model. RecepSinan Arslan [31] also uses a graph convolutional neural network, but the novelty is that it converts the data obtained in AndroidManifest.xml into image data and sends it to the neural network for learning, and its final accuracy rate reaches 96.2%. Shanxi Li [32] et al. first extracted API call sequences from malware code and generated directed cyclic graphs, then used Markov chain and principal component analysis methods to extract feature maps of the graphs and designed a classifier based on graph convolutional networks, and finally achieved 98.32% accuracy. The graph data structure is able to depict and contain more information about the application, providing a sufficient data base for the determination of the nature of the application. Yujie Fan [33] studied how to describe malware and embedded features into heterogeneous networks, and proposed to use metagraph2vector to represent heterogeneous graphs for the first time. The most difficult problem in Android malware detection based on graph neural networks is how to use graph data structures to express Android malware. Although Yujie Fan use heterogeneous graph to express Android malware, there is still a large feature redundancy, resulting in the inability to obtain the optimal feature information.

Commonly used datasets for Android malware detection include Genome dataset [34], Drebin dataset, and AMD dataset [35]. The Genome dataset contains 1260 malware from 2011 to 2012, the Drebin dataset is an extension of the Genome dataset, which contains 5560 malware from 2011 to 2014, the AMD dataset contains 24553 samples from 2010 to 2016. Although the above datasets are rich in information and highly reliable, they are relatively old, emerging malware emerges in an endless stream, and attack methods vary.

The Z2F Android malware detection framework proposed in this article extracts the multinational Android features uses HIN to construct the relationship between different Android features. It combines the graph attention mechanism and the LDA dimensionality reduction algorithm to mine and display the high-order hidden information behind the Android features, removing redundant features to obtain the optimal feature vector, it solves the problem of feature redundancy. To solve the problem of relatively old public datasets, this article collected 20,000 Android applications from 2017 to 2019, and examined a total of 14,429 Android applications.

## 3 Proposed methodology

The macro workflow of Z2F is to extract multidimensional features from the original Android application file, embedding them in heterogeneous graphs then mining the hidden information based on meta-structures, combining the label (benign or malicious) as input to determine the others.

### 3.1 Main methods of Z2F

The key idea of this paper is to use graph neural networks to mine the hidden information behind the features. Since Android applications have a variety of features and different relationships between them, this paper uses heterogeneous graph. The heterogeneous graph data structure *G* = {*V*, *E*, *R*, *T*}, where *V* represents the node set, *E* represents the edge set, *R* represents the type set of the edge, and *T* represents the type set of the node, where [*R*] + [*T*] > 2. In this paper, the original features of text type convert to vector using one-hot encoding, and then embedding in the heterogeneous graph, Android applications and their features are nodes, and the associations between features are edges. As the final object of the detection task is an Android application, it is necessary to update the homogeneous graph with only application nodes based on the constructed heterogeneous graph.

### 3.2 Architecture overview

**Fig 1:**
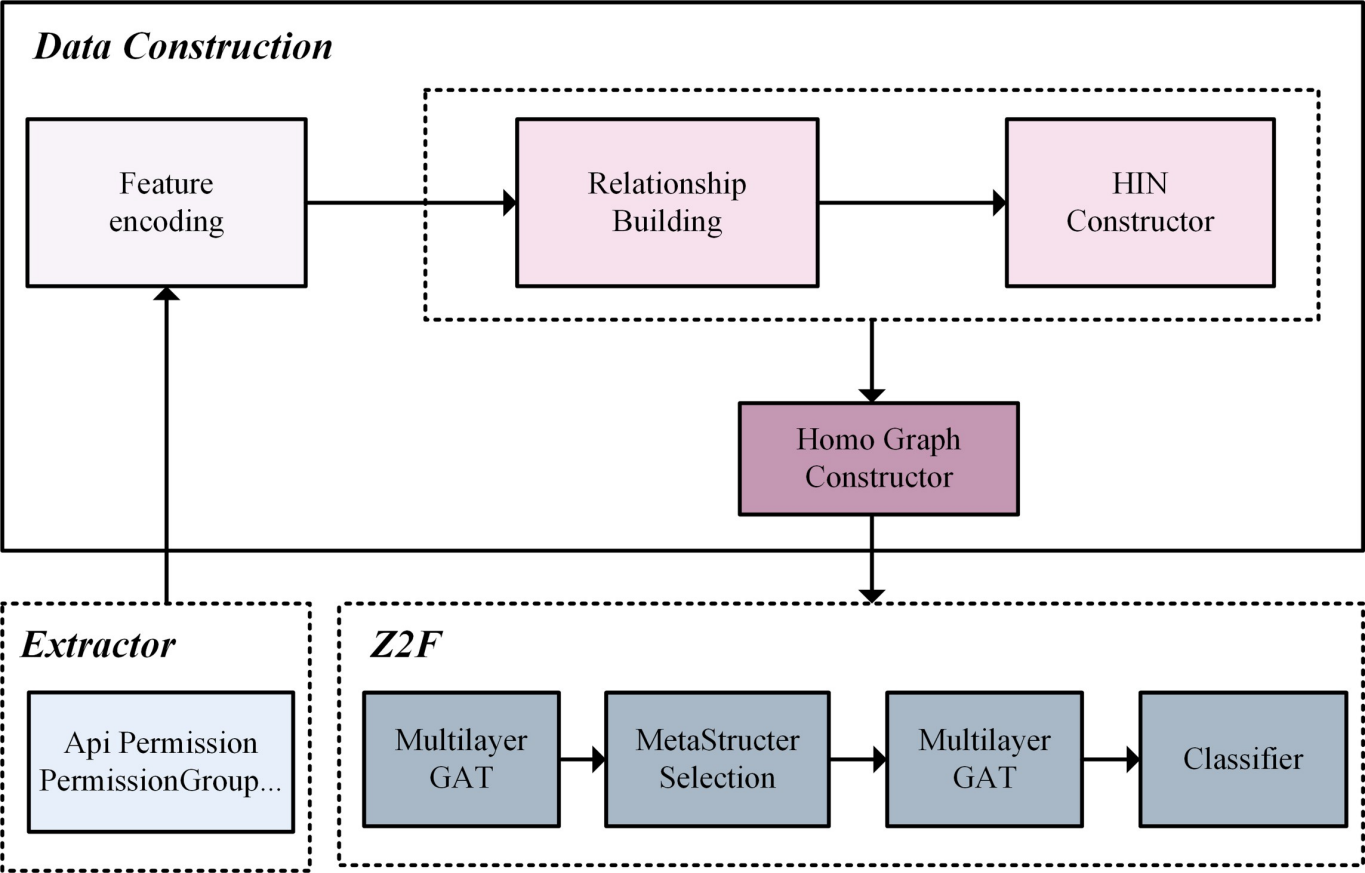
Model architecture diagram.

Fig 1 shows the overall architecture diagram. The feature extraction module decompiles Android application installation packages and extracts features. The feature encoding module using one-hot encoding, on this basis, using the linear discriminant analysis algorithm(LDA) [36] to filter redundant feature information, making the subsequent detection process more lightweight. Heterogeneous graph construction module integrates applications and their features into a heterogram, which is used to initialize the Android application behavior. The homogeneous graph generation module utilizes the heterogeneous graph as a foundation to generate homogeneous subgraphs. This process serves as a precursor for subsequent feeding into the graph neural network and is primarily achieved through the 12 meta-structures we have designed.

Z2F consists of a graph representation learning model using a multi-layer graph attention mechanism. Under the meta-structures we designed, the corresponding homogeneous subgraphs are generated, and the graph attention mechanism is used to perform multi-layer aggregation of meta-structures and iteratively update the final Android application information. Specifically, based on the derived homogeneous graphs, we first use the graph attention mechanism for each of the 12 different meta-structures to fully aggregate the information of neighboring nodes, thus enhancing the representation of the application nodes. In order to make the representation accurate and sufficient, we again use the graph attention mechanism among the selected meta-structures to fully aggregate node information at the semantic level. After completing these two aggregations, the final node embeddings are obtained. Fedding to the classifier module, which uses the final node embeddings information to learn a classification model that detecting malware. The test data is then applied to the classification model to test the performance. The classification model selected in this paper is K-Nearest Neighbor algorithm (KNN).

### 3.3 Data pre-processing

#### 3.3.1 Feature extraction

The Android application is packaged into an .apk file and installed on the Android system, decompiled apk file contains the AndroidManifest.xml, META-INF, classes.dex, res, resources.arsc, smali folder, lib folder and assets folder. The AndroidManifest.xml file stores a large amount of configuration information about the Android application, including the component configuration of the application. The class.dex contains the executable code of the Android application, which is the target when analyzing the application. In this paper, we first use Apktool to decompile the .apk file and generate the disassembly code. By reading the .smali file, we can understand the running mechanism of the Android application and find a breakthrough. After extensive research and domain experts advice, the following seven types of features were extracted for the initial description of Android applications.

**Permission:** Android applications request permissions from the user when accessing restricted data and actions. For example, ACCESS_FINE_LOCATION indicates that the application requests permission to obtain the user’s precise location, and ACCESS_COARSE_LOCATION indicates that the application requests permission to obtain the user’s approximate location. We extract the application’s permission information from the AndroidManifest.xml file.

**Permission group:** Google officially assigns permission groups to all permissions in Android development to ensure that when an application requests multiple closely related permissions from the user, it reduces the number of system dialog prompts. For example, ACCESS_FINE_LOCATION and ACCESS_COARSE_LOCATION all belong to the LOCATION group, and permissions in the same permission group largely reflect related behavior.

**API:** Google officially provides a large number of APIs for the development of Android applications, making it easier to develop Android applications. The API called by the application can directly reflect the behavior of the application. We extract the API calls from the .smali file.

**External package:** Android applications are developed using the Java language, and the function required by the application to achieve a certain behavior can be obtained through .class instruction. At the beginning of the .smali file, the .class instruction specifies the full signature of the class information, and we get the external package from it.

**Parent class:** In the Java language, child classes inherit attributes and methods from their parents, so the two are inextricably linked. At the beginning of the .smali file, the .super instruction specifies the full parent class information.

**Interface:** The interface can clearly reflect a series of methods and functions, allowing the reaearchers to quickly understand what the interface implements and based on this, analyze the behavior of the application. We extract the interface information from the .smali file.

**so file:** The .so file is a dynamic link library file that is used to get the CPU information. The decompiled lib folder holds .so files corresponding to different processor architectures, with the aim of increasing the device compatibility of the application.

#### 3.3.2 Construction of the HIN

**Fig 2:**
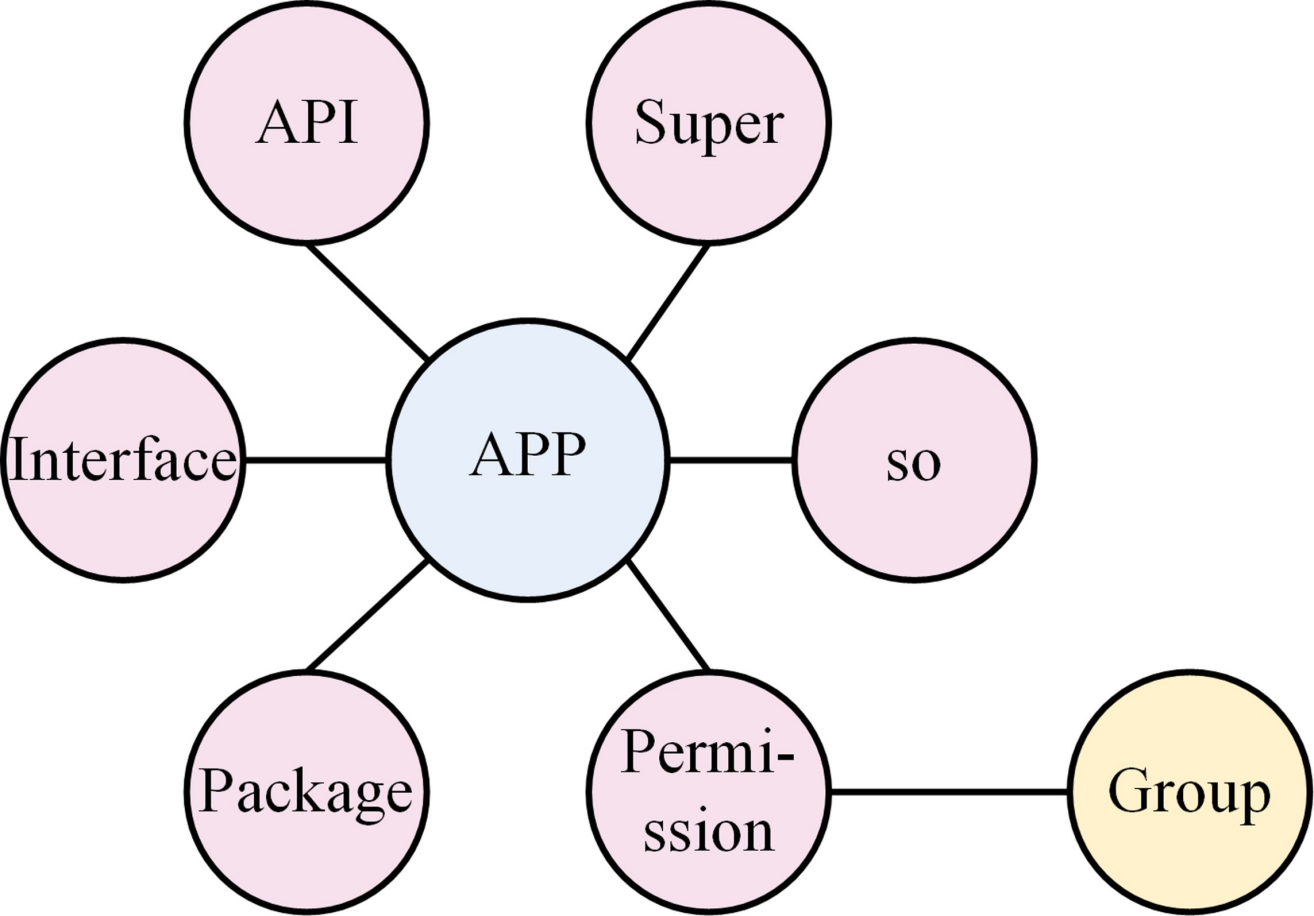
Android application description based on heterogeneous graph.

We use seven types of features introduced above to initially characterize the application behaviors. The relationship between nodes and edges in a heterogeneous graph will be defined as in Fig 2.

**APP-API:** The APP-API describes which APIs are called by an Android application, and the fact that different Android applications call the same APIs indicates that different applications have similar behavior.

**APP-Permission:** Android applications need to apply for permissions from users when they access sensitive data or behaviors. The APP-PERMISSION investigates which permissions Android applications apply for.

**Permission-Permission Group:** Each permission of an Android application belongs to a permission group, different permissions in the same group are highly correlated. Such as READ_EXTERNAL_STORAGE and WRITE_EXTERNAL_STORAGE belong to the STORAGE group. Table 1 lists the specific information about permissions and their groups. If group A includes permission 1, permission 2, and permission 3, then when an APP has applied for permission 1, it does not prompt the user while this app wants to apply the other permissions in the same group. If one application configuration file contains group 1, and another application configuration file also contains group 1, of course they are closely related. Based on this, the potential connection between the two applications was discovered.

**Table 1:** Permission group.

Permission Group	Permission
CALENDAR	READ_CALENDAR, WRITE_CALENDAR
CAMERA	CAMERA
CONTACTS	READ_CONTACTS, WRITE_CONTACTS, GET_ACCOUNTS
LOCATION	ACCESS_FINE_LOCATION, ACCESS_COARSE_LOCATION
MICROPHONE	RECORD_AUDIO
PHONE	READ_PHONE_STATE, CALL_PHONE, READ_CALL_LOG
SENSORS	WRITE_CALL_LOG, ADD_VOICEMAIL, USE_SIP
SMS	PROCESS_OUTGOING_CALLS

**APP-Package:** This relationship indicates external package information called by the Android application during runtime. Many malicious applications call specific packages to achieve malicious behavior.

**APP-Interface:** This describes the use of a specific interface by an application to achieve a specific behavior.

**APP-Super:** This relationship indicates the parent class called by the application to achieve a specific behavior, which is completely recorded in the .smali file.

**APP-.SO:** This indicates the processor information required by the different applications to interact with the hardware device.

**Fig 3:**
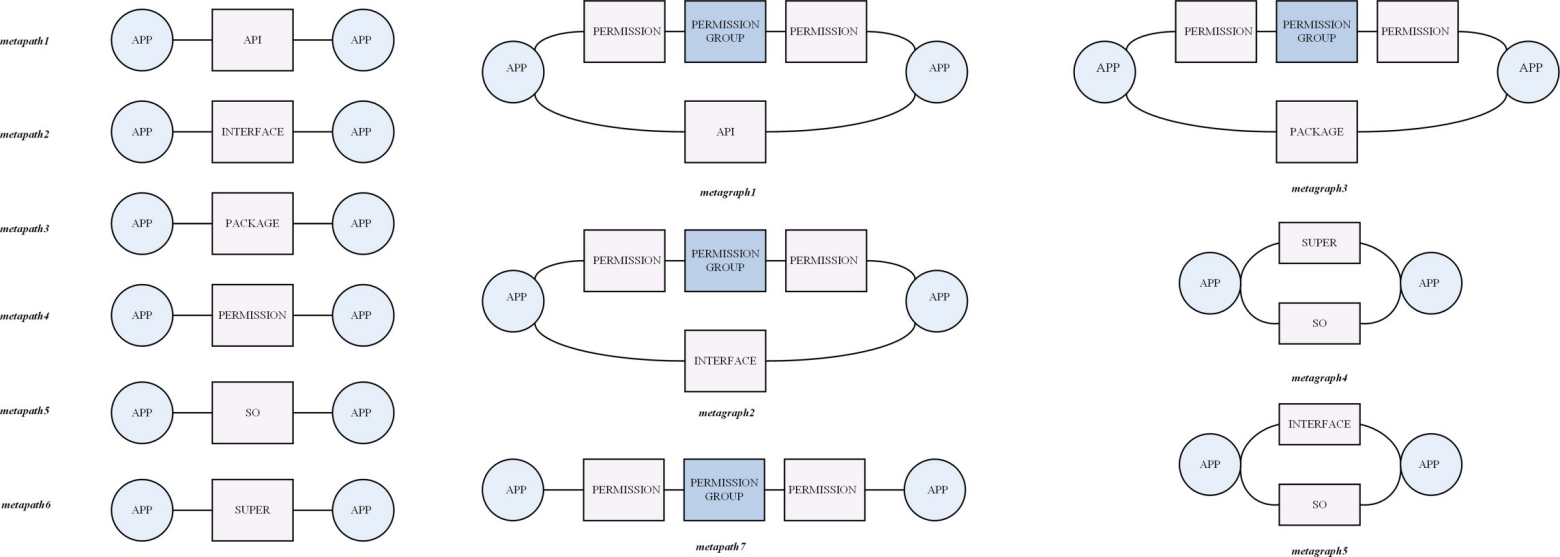
12 kinds of meta-structers.

To explore the hidden relationships behind the features, 12 different meta-structures are designed as in Fig 3, where nodes represent different types of features and edges represent the association relationships between features. The features are embedded according to the predefined meta-structures; based on these 12 meta-structures, we explore the connections between different features and fully exploit the high-order semantic information.

**Fig 4:**
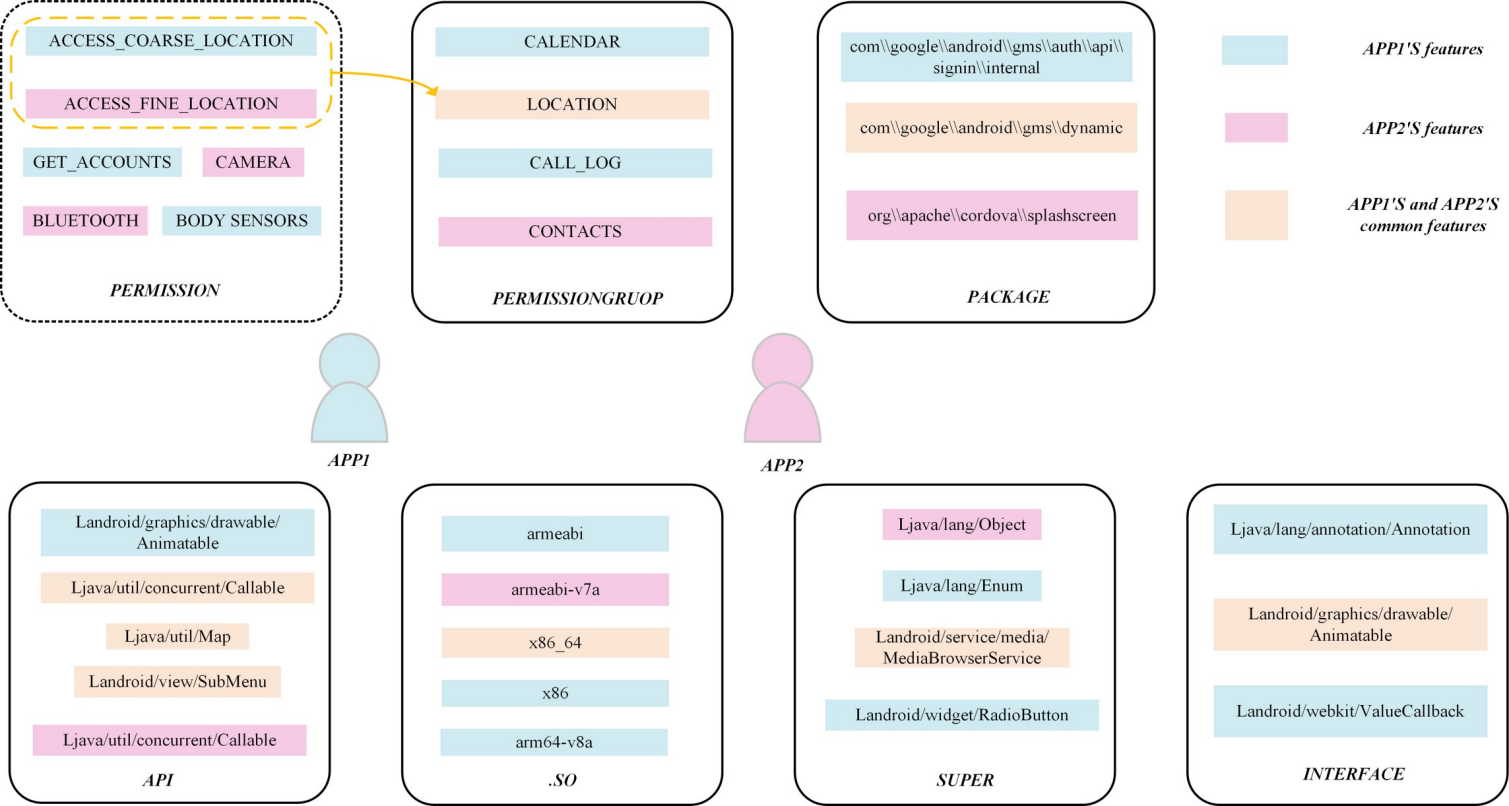
Extracted entity relationships between any two applications.

Fig 4 illustrates the relationship between two applications. We have extracted the ACCESS_FINE_LOCATION permission from application1 and the ACCESS_COARSE_LOCATION permission from application2, which are different but belong to the LOCATION group. Both application1 and application2 contain the “com//google//android//gms//dynamic” external package and both require an x86-64 processor. Two seemingly unrelated applications have been mined lots of contacts.

To generate homogeneous graphs from heterogeneous graphs, the most critical problem is how to link the feature information contained in a single application with other applications. We address this through meta-structures, which are designed to uncover the co-features contained in different applications, and then a homogeneous graph is generated that contains only the different application nodes. At the same time, it reflects the similarity between the different applications.

Unlike homogeneous graph, heterogeneous graph has a variety of node types and edge types, so we use metapaths and metagraphs for mining hidden information on heterogeneous graphs. Metapath connects different types of nodes, and the hidden information of a particular node’s neighboring nodes based on it will be mined. Metagraph are undirected graph that connect different metapaths and contain richer information. Both metapaths and metagraphs can be considered sub-graphs of heterogeneous graphs.

Meta-structure: A metapath represents connections between different features. A metagraph is similar to a metapath, which is a connection of different metapaths, but is more narrowed at the semantic level, and the similar behavior of different applications is more rigorously explored. For example, the metapath APP-PERMISSION-APP retrieves the same permissions that any two applications request, and the metapath APP-PACKAGE-APP indicates that any two applications call the same development package. For example, metagraph1 requires retrieving two applications that both apply the same permissions and call the same APIs.

With the help of metapaths and metagraphs, we derive the corresponding homogeneous graphs from the heterogeneous graph. We use formula (1) to calculate the homogeneous graph matrix for a given metapath as follows:

$$P^{metapath}=M_{f_{1}f_{2}}\cdot M_{f_{2}f_{3}}\cdot M_{f_{3}f_{4}}\cdots \cdot M_{f_{n-1}f_{n}}$$ (1)

where ***f***_***n***_ represents the feature, $M_{f_{n-1}f_{n}}$ represents the relationship between features. Specifically, for the metapath APP-PACKAGE-APP, we use the matrix ***M***_***app-permission***_ to derive the permission information contained in different applications, and then calculate ***M***_***permission-app***_ to indicate which permissions exist in which applications. After deriving ***M***_***app-permission***_ and ***M***_***permission-app***_, the formula (1) can be used to calculate co-permissions in any two apps and the number of co-permissions, by observing, ***M***_***app-permission***_ is a transpose matrix of ***M***_***app-permission***_. In the ***P***^***metapath***^ matrix, any two app nodes are neighbors based on the specific metapath. We can intuitively obtain the similarity of any two apps according to the values in the ***P***^***metapath***^.

Similarly, for a given metagraph, it is formed by connecting two or more metapaths. Therefore, its matrix is given by formula (2) as follows:

$$G^{metagraph}=p^{metapath_{1}}⊙p^{metapath_{2}}⊙p^{metapath_{3}}\cdots ⊙p^{metapath_{n}}$$ (2)

where ⊙ represents the Hadamard product. It can be seen that a metagraph is obtained by multiplying metapaths by Hadamard product. Compared to metapath, metagraph can uncover more hidden information. For example, metagraph1 contains two metapaths, APP-PERMISSION-PERMISSIONGRUOP-PERMISSION-APP and APP-API-APP, which require the same permission request and API call information between any two applications. Its calculation formula is $G^{metagraph_{1}}=p^{metapath_{1}}⊙p^{metapath_{2}}$. According formula (1) and formula (2), we can derive 12 matrices that correspond to each 12 meta-structures.

### 3.4 Node embedding model

**Fig 5:**
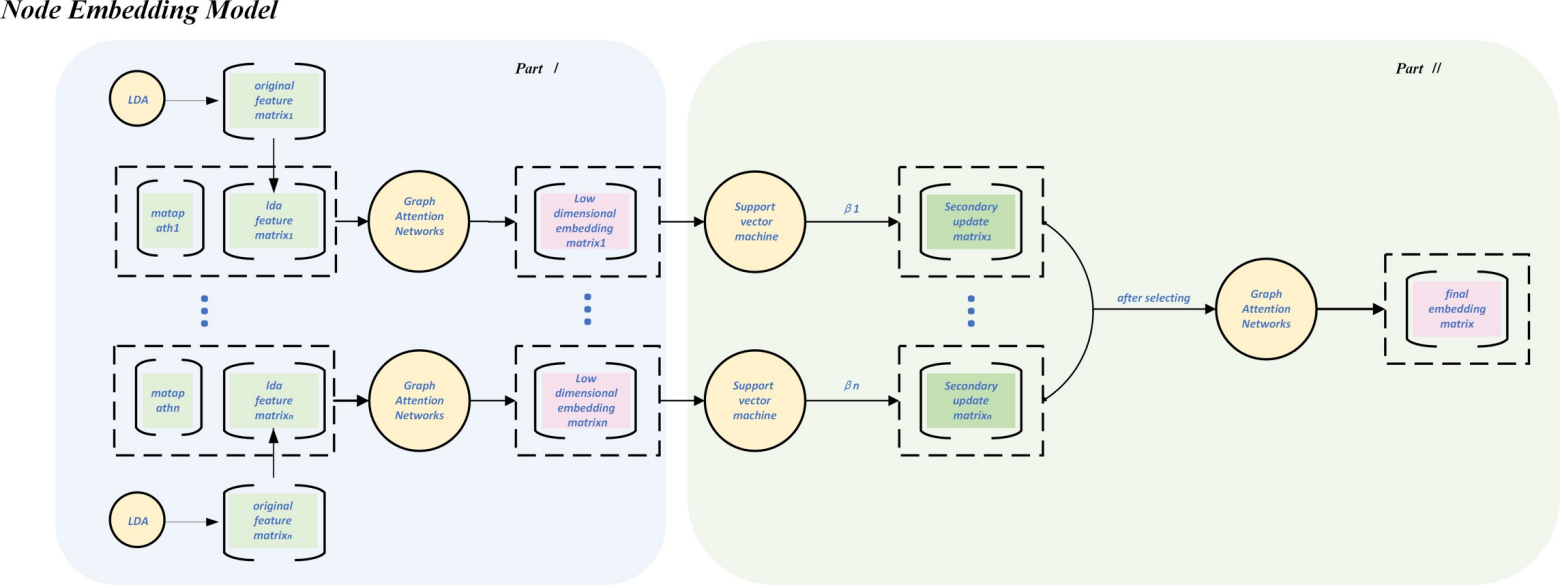
Node embedding model in Z2F.

The node embedding model proposed in this paper is divided into two parts, as shown in Fig 5. In order to fully and comprehensively mine the effective information of neighbor nodes based on the meta-structure, first use the attention mechanism for node aggregation at the specific meta-structure level. Since different meta-structures mean different semantic spaces, we propose a second major part: node aggregation is again performed between different meta-structures using the attention mechanism, which aims to make the information expressed by each node semantically rich and descriptively concise.

Aggregation within a meta-structure is done for a particular meta-structure by first constructing an adjacency matrix of the meta-structure using formula (1) and formula (2). Using the LDA algorithm to reduce the dimensionality of all extracted features, as shown in formula (3).


$$\begin{array}{c} Feature_{di}=LDA(Encoder(api),Encoder(permission),\\ Encoder(permissiontype),Encoder(package),\\ \left(super),Encoder(so),Encoder(interface)\right) \end{array}$$ (3)

***Feature***_***di***_ each row represents one application. The graph attention mechanism(GAT) in the meta-structure focuses on node feature descriptions within the specific semantic space, as shown in formula (4) and formula (5).

$$L^{metapath_{j}}=GAT\left(Feature_{di},p^{metapath_{j}}\right)$$ (4)

$$L^{metagraph_{j}}=GAT\left(Feature_{di},G^{metagraph_{j}}\right)$$ (5)

where *i* ∈ [1,2,3,4,5,6,7], *j* ∈ [1,2,3,4,5,6,7].

After this stage, we derived the low-dimensional embeddings of all samples. Then feed them into a support vector machine(SVM) [37] to obtain the contribution rate of different meta-structures. The meta-structures with a low rate will be eliminated, and the selected meta-structures will be aggregated again using the attention mechanism, which aims at updating the node at the semantic level to obtain an accurate and simplified Android behavior description. The final embedding matrix gives the precise description of each sample in each row.

For selection, the specific operation is as follows: We first use the SVM to learn different meta-structures and obtain the contribution rate, as shown in formula (6).


$$\left(\beta^{metapath_{1}},\cdots ,\beta^{metagraph_{5}}\right)=softmax\left(SVM\left(L^{metapath_{1}}\right),\cdots ,SVM\left(L^{metagraph_{5}}\right)\right)$$ (6)

We select the meta-structures with a higher rate and perform secondary aggregation. The secondary update matrix of nodes is obtained as formula (7). On this basis, we use GAT again to update node information, getting a fuller description of Android behavior.


$$S=\sum_{i=1}^{7}\beta_{i}L_{i}$$ (7)

## 4 Description of datasets

We used AndroZoo to collect 20,000 Android applications with a size of 10M-20M, including 10,000 benign applications and 10,000 malicious applications. First, use the 360 tool to deduplicate all samples, and perform secondary testing on benign samples to ensure that the benign samples are not infected. Secondly, we use the Apktool to decompile all samples. It should be emphasized that some malicious samples are hardened and their real executable files are hidden. These samples are removed from our dataset. After this step, a total of 14,429 samples remained, including 7,239 benign samples and 7,190 malignant samples.

**Fig 6:**
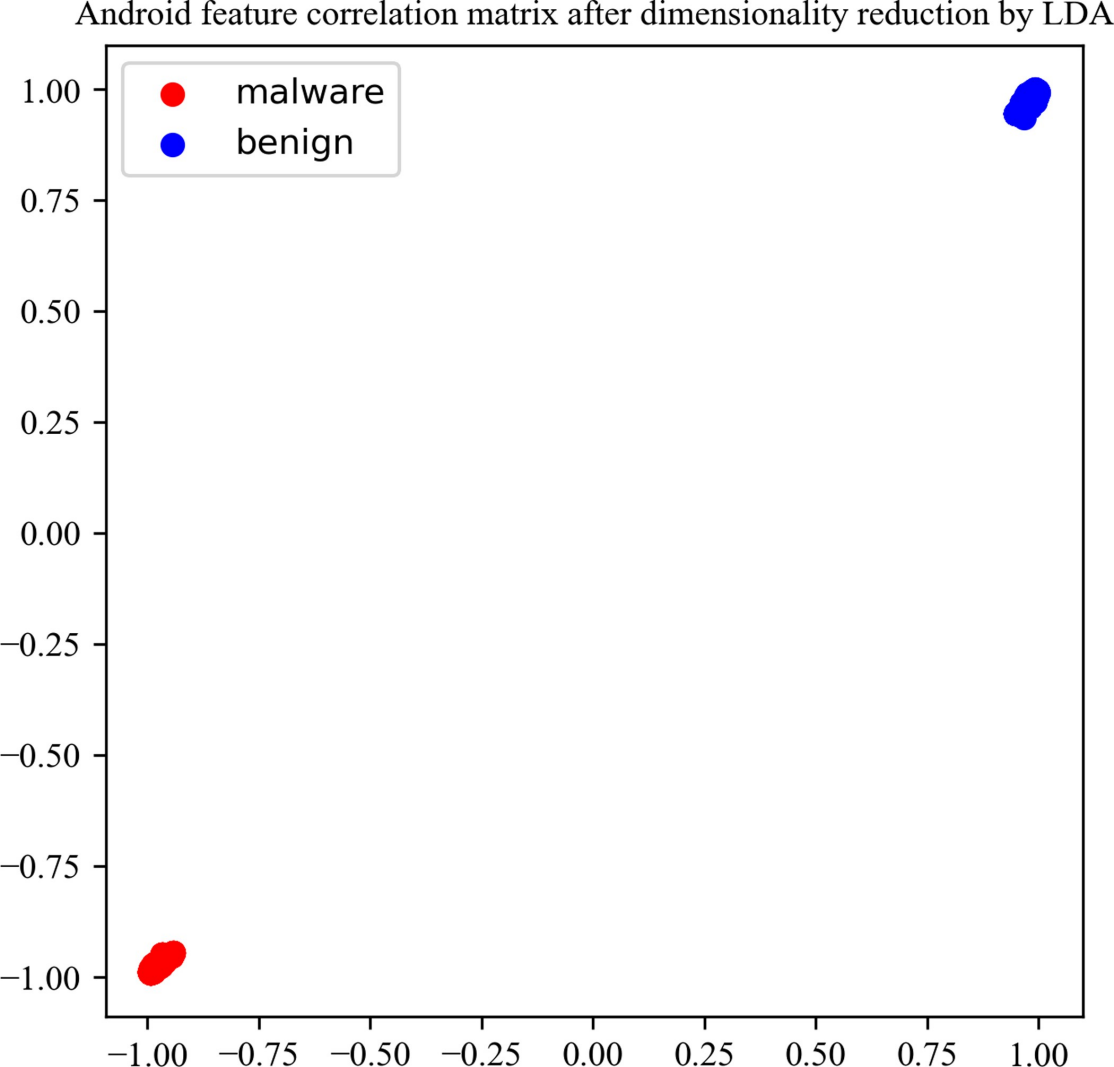
Android feature correlation matrix after dimensionality reduction by LDA.

**Fig 7:**
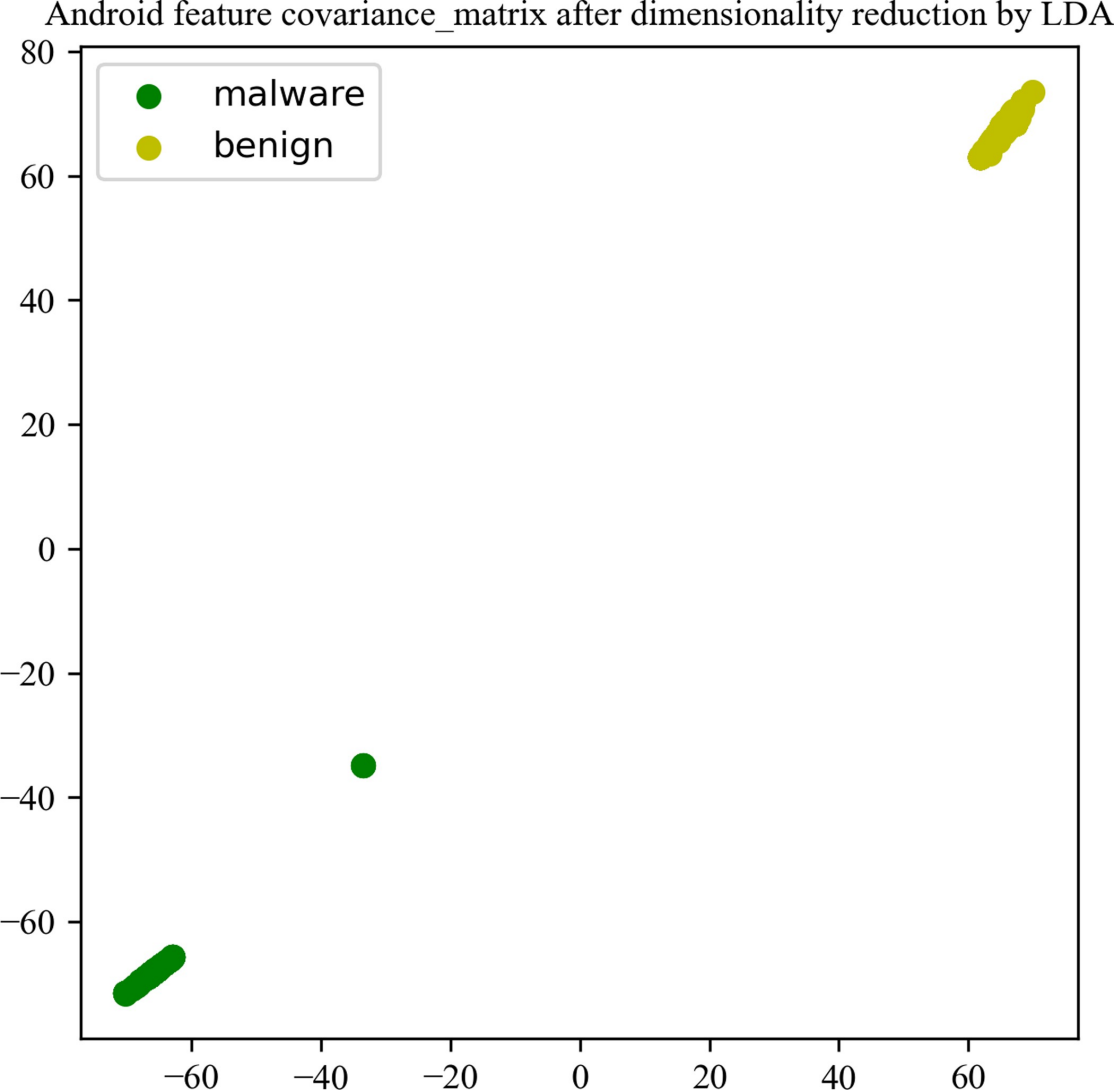
Android feature covariance matrix after dimensionality reduction by LDA.

For each decompiled Android application, the Android features permission, permission groups, api, external package, parent class, Interface, .so are extracted. This article makes statistics on the dimensions of each feature, uses one-hot to encode the original text type features. Due to the existence of redundant features, the one-hot encoded features are dimensionally reduced using the LDA dimensionality reduction algorithm. The dimension statistics of Android key features before and after dimensionality reduction are shown in Table 2. In order to better understand our data, we calculated the correlation and covariance of the features after dimensionality reduction and visualized them, as shown in Figs 6 and 7. Through the correlation matrix and covariance matrix, we can observe that LDA has a significant dimensionality reduction effect, which plays a strong supporting role in subsequent Android malware detection. The covariance matrix reflects the dispersion phenomenon among malignant samples. We analyzed this and concluded that it is due to the different types of malware in the samples.

**Table 2:** Comparison of dimensions before and after LDA reduction.

dimension features	after one-hot	after LDA
Api	765794	10
Interface	91278	10
Package	49167	10
Permission	341	10
Permissiongroup	10	10
So	28949	10
Super	103856	10

## 5 Results and discussion

### 5.1 Experimental environment

Firstly, under the 11th Gen Intel(R) Core(TM) i5-12400 16GB RAM Windows 10, the collected benign samples were scanned and detected using 360 Antivirus to ensure purity, and all samples were de-duplicated using 360 Security Guard. The Android application was decompiled using Apktool 2.0 and JDK 1.8. The proposed model was trained using Ubuntu 18.04 with a Linux kernel, tensorflow-gpu framework and a GPU configuration of an RTX 3080. In Table 3, our model’s all hyperparameters are listed.

**Table 3:** Our model’s hyperparameters.

Component	Parameter	Value
**GAT**	hidden layers	8
	heads	16
	output length	64
**Training**	learning rate	0.005
	batch size	1
	weight decay	0.001
	activation function	elu

### 5.2 Datasets

In this paper, a total of 20,000 Android applications were collected with the help of AndroZoo, all benign samples were from the Google App Store, and all malicious samples were from VirusShare. Since the size of the collected samples is 10M-20M, through sample distribution experiment, we use stratified sampling method to divide the 14429 samples into 7 sub-datasets named DB1-DB7 respectively. In each sub-dataset, 10%, 20%, 30%, and 40%. 50%, 60%, 70%, and 80% of the samples are applied to train the model, and the remaining samples are used for testing.

### 5.3 Metrics

Based on confusion matrix for two-class classification as Table 4, we use the accuracy as formula (8) and F1 score as formula (11) to evaluate the model’s effectiveness, “accuracy” refers to the ratio of the number of samples predicted correctly by the model to the total number of samples. The F1 score as formula (11) is an indicator that comprehensively considers recall see formula(9) and precision see formula (10). It is the harmonic average of these two indicators. It can consider the accuracy and coverage of the model at the same time. The average accuracy using 10-fold cross-validation to evaluate our classifier.

**Table 4:** Confusion matrix for two-class classification.

Actual/Predicted as	Positive	Negative
**Positive**	TP	FN
**Negative**	FP	TN


$$accuracy=\frac{TP+TN}{TP+FN+FP+TN}$$ (8)

$$recall=\frac{TP}{TP+FN}$$ (9)

$$precision=\frac{TP}{TP+FP}$$ (10)

$$F1=\frac{2\times precision\times recall}{precision+recall}$$ (11)

### 5.4 Baseline

In order to evaluate the performance of Z2F, this article uses 3 representative methods as baselines for comparison. Daniel Arp used extensive static analysis to embed features in a joint vector space and used the machine learning method SVM. Cagatay Catal [38]used an application programming interface (API) call graph obtained from malware and benign Android apk files to solve the Android malware detection problem using a graph attention network model. Han Gao [39] proposed a new method for detecting Android malware based on the graph convolutional neural network model.

### 5.5 Results and discussion

The accuracy and F1 scores of the different methods are shown in Tables 5 and 6. We can see that our proposed Z2F model achieves a significant improvement in classification accuracy. Compared to other methods, which relies too much on the Android features but ignores the connection behind them, Z2F explore the hidden information behind Android features at semantic level, the meta-structures and multilayer attention mechanism of our proposed Z2F model provides powerful support.

**Table 5:** The F1 value of sample apps detection.

	dataset	Model	10%	20%	30%	40%	50%	60%	70%	80%
**F1**	**DB1**	**Daniel [3]**	0.8832	0.8886	0.8928	0.9075	0.9389	0.9300	0.9466	0.9500
		**Cagatay [38]**	0.8323	0.8368	0.8394	0.8445	0.8507	0.8660	0.8693	0.8725
		**Han [39]**	0.8154	0.8269	0.8393	0.8332	0.8324	0.8349	0.8383	0.8455
		**Z2F**	**0.9971**	**0.9971**	**0.9974**	**0.9976**	**0.9973**	**0.9977**	**0.9974**	**0.9979**
	**DB2**	**Daniel [3]**	0.8696	0.9055	0.9200	0.9400	0.9418	0.9474	0.9283	0.9449
		**Cagatay [38]**	0.8356	0.8377	0.8391	0.8598	0.8631	0.8695	0.8718	0.8738
		**Han [39]**	0.8088	0.8122	0.8189	0.8444	0.8463	0.8409	0.8501	0.8501
		**Z2F**	**0.9970**	**0.9974**	**0.9979**	**0.9969**	**0.9977**	**0.9979**	**0.9976**	**0.9975**
	**DB3**	**Daniel [3]**	0.8672	0.8911	0.9135	0.9200	0.9250	0.9250	0.9280	0.9174
		**Cagatay [38]**	0.8306	0.8331	0.8485	0.8501	0.8516	0.8549	0.8579	0.8611
		**Han [39]**	0.8284	0.8251	0.8287	0.8284	0.8382	0.8372	0.8414	0.8586
		**Z2F**	**0.9935**	**0.9934**	**0.9931**	**0.9945**	**0.9950**	**0.9934**	**0.9940**	**0.9953**
	**DB4**	**Daniel [3]**	0.9093	0.9231	0.9250	0.9340	0.9450	0.9411	0.9515	0.9499
		**Cagatay [38]**	0.8348	0.8393	0.8429	0.8450	0.8669	0.8760	0.8732	0.8691
		**Han [39]**	0.8020	0.8217	0.8228	0.8399	0.8500	0.8592	0.8428	0.8500
		**Z2F**	**0.9984**	**0.9987**	**0.9984**	**0.9983**	**0.9981**	**0.9982**	**0.9988**	**0.9996**
	**DB5**	**Daniel [3]**	0.8496	0.8837	0.9043	0.9317	0.9360	0.9335	0.9350	0.9575
		**Cagatay [38]**	0.8339	0.8443	0.8431	0.8408	0.8583	0.8482	0.8582	0.8535
		**Han [39]**	0.8001	0.8338	0.8349	0.8356	0.8477	0.8404	0.8673	0.8578
		**Z2F**	**0.9979**	**0.9981**	**0.9978**	**0.9975**	**0.9970**	**0.9968**	**0.9962**	**0.9986**
	**DB6**	**Daniel [3]**	0.8754	0.9162	0.9393	0.9342	0.9380	0.9487	0.9400	0.9525
		**Cagatay [38]**	0.8234	0.8383	0.8327	0.8481	0.8440	0.8420	0.8400	0.8394
		**Han [39]**	0.8153	0.8223	0.8310	0.8303	0.8420	0.8552	0.8523	0.8540
		**Z2F**	**0.9966**	**0.9968**	**0.9974**	**0.9968**	**0.9971**	**0.9979**	**0.9976**	**0.9996**
	**DB7**	**Daniel [3]**	0.8772	0.8831	0.8843	0.9175	0.9130	0.9267	0.9380	0.9475
		**Cagatay [38]**	0.8330	0.8518	0.8491	0.8452	0.8411	0.8381	0.8372	0.8397
		**Han [39]**	0.8111	0.8174	0.8209	0.8385	0.8419	0.8461	0.8525	0.8634
		**Z2F**	**0.9907**	**0.9919**	**0.9945**	**0.9924**	**0.9943**	**0.9959**	**0.9974**	**0.9971**

**Table 6:** The accuracy value of sample apps detection.

	dataset	Model	10%	20%	30%	40%	50%	60%	70%	80%
**acc**	**DB1**	**Daniel [3]**	0.8833	0.8888	0.8929	0.9075	0.9390	0.9300	0.9467	0.9500
		**Cagatay [38]**	0.8324	0.8367	0.8395	0.8446	0.8508	0.8661	0.8694	0.8725
		**Han [39]**	0.8155	0.8269	0.8393	0.8333	0.8325	0.8348	0.8384	0.8456
		**Z2F**	**0.9971**	**0.9971**	**0.9974**	**0.9976**	**0.9973**	**0.9977**	**0.9974**	**0.9979**
	**DB2**	**Daniel [3]**	0.8700	0.9056	0.9200	0.9400	0.9420	0.9475	0.9283	0.9450
		**Cagatay [38]**	0.8356	0.8376	0.8390	0.8598	0.8630	0.8695	0.8712	0.8733
		**Han [39]**	0.8082	0.8129	0.8184	0.8447	0.8463	0.8408	0.8501	0.8500
		**Z2F**	**0.9970**	**0.9974**	**0.9979**	**0.9969**	**0.9977**	**0.9979**	**0.9976**	**0.9975**
	**DB3**	**Daniel [3]**	0.8672	0.8911	0.9136	0.9200	0.9250	0.9250	0.9283	0.9175
		**Cagatay [38]**	0.8307	0.8332	0.8480	0.8502	0.8511	0.8546	0.8578	0.8610
		**Han [39]**	0.8281	0.8252	0.8286	0.8286	0.8383	0.8373	0.8415	0.8587
		**Z2F**	**0.9935**	**0.9934**	**0.9931**	**0.9945**	**0.9950**	**0.9934**	**0.9940**	**0.9954**
	**DB4**	**Daniel [3]**	0.9094	0.9231	0.9250	0.9342	0.9450	0.9413	0.9517	0.9500
		**Cagatay [38]**	0.8347	0.8393	0.8428	0.8451	0.8668	0.8761	0.8731	0.8692
		**Han [39]**	0.8021	0.8216	0.8227	0.8398	0.8503	0.8591	0.8427	0.8501
		**Z2F**	**0.9984**	**0.9987**	**0.9984**	**0.9983**	**0.9981**	**0.9982**	**0.9988**	**0.9996**
	**DB5**	**Daniel [3]**	0.8500	0.8838	0.9043	0.9317	0.9360	0.9337	0.9350	0.9575
		**Cagatay [38]**	0.8338	0.8442	0.8432	0.8407	0.8582	0.8481	0.8581	0.8534
		**Han [39]**	0.8001	0.8337	0.8348	0.8355	0.8478	0.8405	0.8673	0.8579
		**Z2F**	**0.9979**	**0.9981**	**0.9978**	**0.9975**	**0.9970**	**0.9968**	**0.9962**	**0.9986**
	**DB6**	**Daniel [3]**	0.8756	0.9162	0.9393	0.9342	0.9380	0.9487	0.9400	0.9525
		**Cagatay [38]**	0.8233	0.8382	0.8326	0.8481	0.8447	0.8421	0.8400	0.8391
		**Han [39]**	0.8154	0.8222	0.8311	0.8301	0.8421	0.8557	0.8523	0.8541
		**Z2F**	**0.9966**	**0.9968**	**0.9974**	**0.9968**	**0.9971**	**0.9979**	**0.9976**	**0.9996**
	**DB7**	**Daniel [3]**	0.8772	0.8831	0.8843	0.9175	0.9130	0.9267	0.9380	0.9475
		**Cagatay [38]**	0.8329	0.8517	0.8490	0.8453	0.8412	0.8380	0.8372	0.8398
		**Han [39]**	0.8110	0.8173	0.8208	0.8384	0.8418	0.8460	0.8524	0.8633
		**Z2F**	**0.9907**	**0.9919**	**0.9945**	**0.9924**	**0.9943**	**0.9959**	**0.9974**	**0.9971**

We evaluated the contribution of 12 meta-structures, and the results are shown in Table 7. The experimental results show that the metapath7 has little contribution to detection. Finally, according to contribution rate, we select 4 metapaths and 3 metagraphs to analyze heterogeneous graphs.

**Table 7:** The average contribution rate of different meta structures.

metastructer	contribution rate	reorder
**metapath1**	0.9409	④
**metapath2**	0.8917	-
**metapath3**	0.9401	⑤
**metapath4**	0.9562	②
**metapath5**	0.8839	-
**metapath6**	0.9481	③
**metapath7**	0.5323	-
**metagraph1**	0.9724	①
**metagraph2**	0.7424	-
**metagraph3**	0.9202	⑦
**metagraph4**	0.8743	-
**metagraph5**	0.9341	⑥

Although our proposed approach has achieved satisfactory results in solving the Android malware problem, it still not completely solves it. We analyze the reasons as follows: in order to resist malicious elements, the developers have used obfuscation and reinforcement protection during the development of the application. After Apktool decompiles, a small part of the Android feature information is difficult to restore to the original field, which makes the training data noisy.

### 5.6 Ablation study

In order to determine the performance of the different components, different modules are evaluated separately, and the following ablation experiments are identified:

I: The first attention mechanism was removed, namely the attention mechanism inside the meta-structures is removed. The evaluation results are shown in Table 8.

**Table 8:** The F1 and accuracy value of sample apps detection.

	dataset	Model	10%	20%	30%	40%	50%	60%	70%	80%
**F1**	**DB1**	**Z2F-I**	0.9285	0.9285	0.9286	0.9285	0.9285	0.9287	0.9283	0.9293
		**Z2F-II**	0.9623	0.9655	0.9666	0.9687	0.9689	0.9695	0.9679	0.9686
		**Z2F**	**0.9971**	**0.9971**	**0.9974**	**0.9976**	**0.9973**	**0.9977**	**0.9974**	**0.9979**
	**DB2**	**Z2F-I**	0.9223	0.9228	0.9232	0.9264	0.9258	0.9266	0.9284	0.9284
		**Z2F-II**	0.9696	0.9699	0.9695	0.9695	0.9697	0.9696	0.9695	0.9693
		**Z2F**	**0.9970**	**0.9974**	**0.9979**	**0.9969**	**0.9977**	**0.9979**	**0.9976**	**0.9975**
	**DB3**	**Z2F-I**	0.9292	0.9292	0.9292	0.9295	0.9297	0.9295	0.9290	0.9290
		**Z2F-II**	0.9617	0.9653	0.9647	0.9652	0.9660	0.9648	0.9652	0.9661
		**Z2F**	**0.9935**	**0.9934**	**0.9931**	**0.9945**	**0.9950**	**0.9934**	**0.9940**	**0.9953**
	**DB4**	**Z2F-I**	0.9275	0.9275	0.9200	0.9200	0.9250	0.9200	0.9275	0.9225
		**Z2F-II**	0.9629	0.9621	0.9632	0.9644	0.9654	0.9652	0.9671	0.9964
		**Z2F**	**0.9984**	**0.9987**	**0.9984**	**0.9983**	**0.9981**	**0.9982**	**0.9988**	**0.9996**
	**DB5**	**Z2F-I**	0.9200	0.9200	0.9225	0.9200	0.9250	0.9200	0.9225	0.9225
		**Z2F-II**	0.9570	0.9640	0.9629	0.9698	0.9644	0.9662	0.9691	0.9624
		**Z2F**	**0.9979**	**0.9981**	**0.9978**	**0.9975**	**0.9970**	**0.9968**	**0.9962**	**0.9986**
	**DB6**	**Z2F-I**	0.9292	0.9292	0.9290	0.9289	0.9280	0.9279	0.9264	0.9271
		**Z2F-II**	0.9694	0.9695	0.9698	0.9698	0.9693	0.9696	0.9696	0.9696
		**Z2F**	**0.9966**	**0.9968**	**0.9974**	**0.9968**	**0.9971**	**0.9979**	**0.9976**	**0.9996**
	**DB7**	**Z2F-I**	0.9249	0.9222	0.9299	0.9274	0.9247	0.9224	0.9299	0.9223
		**Z2F-II**	0.9692	0.9696	0.9692	0.9693	0.9691	0.9693	0.9698	0.9689
		**Z2F**	**0.9907**	**0.9919**	**0.9945**	**0.9924**	**0.9943**	**0.9959**	**0.9974**	**0.9971**
**acc**	**DB1**	**Z2F-I**	0.9285	0.9285	0.9285	0.9285	0.9283	0.9288	0.9283	0.9293
		**Z2F-II**	0.9623	0.9655	0.9666	0.9687	0.9689	0.9695	0.9679	0.9686
		**Z2F**	**0.9971**	**0.9971**	**0.9974**	**0.9976**	**0.9973**	**0.9977**	**0.9974**	**0.9979**
	**DB2**	**Z2F-I**	0.9223	0.9228	0.9232	0.9232	0.9258	0.9266	0.9284	0.9284
		**Z2F-II**	0.9696	0.9699	0.9695	0.9695	0.9697	0.9696	0.9695	0.9693
		**Z2F**	**0.9970**	**0.9974**	**0.9979**	**0.9969**	**0.9977**	**0.9979**	**0.9976**	**0.9975**
	**DB3**	**Z2F-I**	0.9292	0.9292	0.9292	0.9295	0.9297	0.9295	0.9290	0.9296
		**Z2F-II**	0.9617	0.9653	0.9647	0.9652	0.9660	0.9648	0.9652	0.9661
		**Z2F**	**0.9935**	**0.9934**	**0.9931**	**0.9945**	**0.9950**	**0.9934**	**0.9940**	**0.9953**
	**DB4**	**Z2F-I**	0.9250	0.9225	0.9275	0.9200	0.9225	0.9250	0.9225	0.9275
		**Z2F-II**	0.9629	0.9621	0.9632	0.9644	0.9654	0.9652	0.9671	0.9664
		**Z2F**	**0.9984**	**0.9987**	**0.9984**	**0.9983**	**0.9981**	**0.9982**	**0.9988**	**0.9996**
	**DB5**	**Z2F-I**	0.9200	0.9225	0.9225	0.9275	0.9200	0.9200	0.9225	0.9250
		**Z2F-II**	0.9570	0.9640	0.9630	0.9698	0.9644	0.9662	0.9691	0.9625
		**Z2F**	**0.9979**	**0.9981**	**0.9978**	**0.9975**	**0.9970**	**0.9968**	**0.9962**	**0.9986**
	**DB6**	**Z2F-I**	0.9292	0.9292	0.9290	0.9289	0.9280	0.9279	0.9264	0.9271
		**Z2F-II**	0.9694	0.9695	0.9698	0.9698	0.9693	0.9696	0.9696	0.9696
		**Z2F**	**0.9966**	**0.9968**	**0.9974**	**0.9968**	**0.9971**	**0.9979**	**0.9976**	**0.9996**
	**DB7**	**Z2F-I**	0.9274	0.9224	0.9249	0.9224	0.9299	0.9224	0.9223	0.9248
		**Z2F-II**	0.9692	0.9696	0.9692	0.9693	0.9691	0.9693	0.9698	0.9689
		**Z2F**	**0.9907**	**0.9919**	**0.9945**	**0.9924**	**0.9943**	**0.9959**	**0.9974**	**0.9971**

II: Removing the second attention mechanism, namely the attention mechanism between selected meta-structures was removed. The evaluation results are shown in Table 8.

## 6 Conclusions and future work

**Expressibility:** The Z2F model extracts a large amount of Android features data from the original Android application files, embedding them into heterogeneous graphs, mining the hidden information based on the 12 meta-structures, and uses a multi-layer graph attention network to iteratively update the node information, which is semantically rich and concise after aggregation by the multi-layer graph attention network. Based on the information mining of graph neural networks, the Z2F model is significantly more accurate for malware detection.

**Scalability:** The Z2F model is based on heterogeneous graphs and meta-structures of Android applications that are designed for cross-temporal by decompiling, extracting, and constructing heterogeneous graphs according to the method we proposed, we can detect Android maware from benign. It is important to note that the official Android API provided by Google is constantly being upgraded, so the compatible version of Apktool needs to be selected when decompiling Android applications. In summary, the model proposed in this paper has excellent scalability, time-detectable.

With the increasing popularity and integration of smart devices, malware is becoming more common, causing data leakage and financial loss to users. Malware detection is an urgent task to reduce the damage at the source. The proposed method in this paper combines Android features with deep learning graph neural networks.

In future work, we will consider the quick detection of new samples (outside the datasets) and develop it as a mobile application for the majority of Android users to install on their mobile devices, so that they can detect the installed applications in the first time and prevent malicious programs from causing losses.

## Supporting information

S1 File Dataset and code. (DOCX)
