# Supplementary material for: Z2F: Heterogeneous graph-based Android malware detection
Source: PLoS One. 2024 Mar 28;19(3):e0300975. doi: 10.1371/journal.pone.0300975 (PMC10977757; doi:10.1371/journal.pone.0300975)
Supplement: S1 File — (DOCX) [file pone.0300975.s001.docx]

This article makes all data and code public in: <https://github.com/Jully-xiaoman/Z2F>
